# Five gene signatures were identified in the prediction of overall survival in resectable pancreatic cancer

**DOI:** 10.1186/s12893-020-00856-y

**Published:** 2020-09-17

**Authors:** Chao Wu, Zuowei Wu, Bole Tian

**Affiliations:** Department of Pancreatic Surgery, West China Hospital, Sichuan University, No. 37 Guoxue Alley, Chengdu, Sichuan Province China

**Keywords:** Pancreatic cancer, Prognostic model, TCGA, Biomarkers, Survival, Nomogram

## Abstract

**Background:**

Although genes have been previously detected in pancreatic cancer (PC), aberrant genes that play roles in resectable pancreatic cancer should be further assessed.

**Methods:**

Messenger RNA samples and clinicopathological data corrected with PC were downloaded from The Cancer Genome Atlas (TCGA). Resectable PC patients were randomly divided into a primary set and a validation set. Univariable Cox regression analysis, lasso-penalized Cox regression analysis, and multivariable Cox analysis were implemented to distinguish survival-related genes (SRGs). A risk score based on the SRGs was calculated by univariable Cox regression analysis. A genomic-clinical nomogram was established by integrating the risk score and clinicopathological data to predict overall survival (OS) in resectable PC.

**Results:**

Five survival-related genes (AADAC, DEF8, HIST1H1C, MET, and CHFR) were significantly correlated with OS in resectable PC. The resectable PC patients, based on risk score, were sorted into a high-risk group that showed considerably unfavorable OS (*p* < 0.001) than the low-risk group, in both the primary set and the validation set. The concordance index (C-index) was calculated to evaluate the predictive performance of the nomogram were respectively in the primary set [0.696 (0.608–0.784)] and the validation set [0.682 (0.606–0.758)]. Additionally, gene set enrichment Analysis discovered several meaningful enriched pathways.

**Conclusion:**

Our study identified five prognostic gene biomarkers for OS prediction and which facilitate postoperative molecular target therapy for the resectable PC, especially the nomic-clinical nomogram which may be used as an effective model for the postoperative OS evaluation and also an optimal therapeutic tool for the resectable PC.

## Introduction

Pancreatic cancer is a malignant neoplasm with a high incidence and mortality worldwide [1, 2]. The near inability to diagnose early stage pancreatic cancer results in missed chances of surgery, which is the main method of cure for PC patients [3]. Additionally, therapeutic schedules for advanced stage pancreatic cancer have made little progress in past decades due to its characteristic insensitivity to radiotherapy and chemotherapy [4]. Furthermore, some of its characteristic proteins are also expressed in chronic pancreatitis, decreasing the biomarker specificity for the diagnosis of PC [5]. Thus, neither the diagnosis nor the therapeutic schedule of pancreatic cancer is satisfactory at present. Therefore, there is an urgent need to identify a novel advanced biomarker for diagnosing and developing therapies for PC patients to decrease mortality. Patients with PC sometimes have very different OS and have different reactions to certain drugs, so key genes that affect OS must be identified.

The TCGA is a National Cancer Institute initiative that provides information on approximately thirty different tumor types with genomic platforms and free access to all investigators [6]. For instance, the mRNA sequencing data and clinicopathological data associated with PC can be obtained from the TCGA.

Accumulating studies have indicated that aberrant genes play important roles in the initiation, progression, and prognosis of tumors and have been explored as cancer biomarkers in past decades [7–12]. Although these aberrant gene have been utilized to establish predictive models for PC prognosis prediction, no consensus on their effectiveness has yet been reached. For instance, Song [13] and Shi [14] established predictive models that only used aberrant gene expression. Song W and colleagues [15] established predictive models for PC prognosis prediction using only clinicopathological data. No model to date has been established based on both aberrant gene data and clinicopathological data. Therefore, in this study, we aimed to identify significant aberrant genes and clinicopathological data that could be used to establish a genomic-clinical nomogram, which in turn to predict the prognosis of PC and develop an optimal therapeutic schedule for resectable PC patients.

## Methods

### Data collection and processing

We downloaded messenger RNA samples and clinicopathological data associated with PC from The Cancer Genome Atlas (https://cancergenome.nih.gov/). We also omitted patients who had zero days of survival, no surgical treatment information, incomplete clinical information, and no gene expression information. Finally, one hundred and twenty-one patients with clinicopathological data associated with messenger RNA information were included, and confirmed by postoperative pathology verified to be pancreatic ductal adenocarcinoma, and were complete resected, Furthermore, patients after surgery were given gemcitabine and 5-fluorouracil chemotherapy. To perform further analysis, the one hundred and twenty-one patients were randomly sorted into a primary set and a validation set.

### Identification of aberrantly expressed mRNA

Transcripts per million normalization and log2 transformation were used for the expression profiles. A total of 14,396 annotated genes were employed for differentially expressed analyses by the “Limma” version 3.6.2 R package [16]. Aberrantly expressed genes were selected for subsequent prognostic analysis if (a) they showed consistent expression patterns in the primary set and (b) they were listed in the validation set.

### Distinguishing the aberrantly expressed mRNA correlated with prognosis

Univariable Cox regression analysis, lasso-penalized Cox regression analysis [17], and multivariable Cox analysis were applied to determine the survival-related genes (SRGs). *P* < 0.01 in the univariable Cox regression analysis was regarded as statistically significant. The SRGs were explored as risk score = (Coefficient mRNA1 × expression of mRNA1) + (Coefficient mRNA2 × expression of mRNA2) + (Coefficient mRNAn × expression mRNAn). The R packages “survival” and “survminer” were applied to evaluate the optimal cutoff of risk score. The performance of the risk score was evaluated by area under the curve (AUC) and survival analysis. The R package “survivalROC” was applied to evaluate the prognostic value of the SRGs [18]. A two-sided log-rank *p* < 0.05 was regarded as significant for survival analysis.

### Identification of clinicopathological data in the primary set and validation set

The clinical information associated with the one hundred and twenty-one PC patients were incorporated into the subsequent analysis. Whole clinical information including age, surgery method, whether radiation, gender, grade stage, and TNM stage were screened to build prognostic model. Multivariable Cox regression analyses were implemented for both the primary set and the validation set. *P* < 0.05 was regarded as statistically significant.

### Development and discriminatory validity of the predictive nomogram

A predictive nomogram was established based on the risk scores and clinicopathological data using a backward stepwise Cox proportional hazard model [19] in both the primary set and validation set. The C-index was used to assess the discriminability of the nomogram [20, 21]. A calibration curve was used to evaluate the performance of the nomogram. The protein levels of the five aberrantly expressed SRGs were validated by The Human Protein Atlas database (http://www.proteinatlas.org).

### Gene set enrichment analyses

To uncover the underlying Kyoto Encyclopedia of Genes and Genomes (KEGG) pathways of the gene signature, gene set enrichment analyses [22] were employed for screening enriched terms in the primary set or validation set. *P* < 0.05 and a false discovery rate q < 0.25 were considered statistically significant.

### Statistical analysis

Statistical analyses were performed using SPSS V21.0 (SPSS Inc.) and R software v3.6.2 (R Foundation for Statistical Computing, Vienna, Austria). Fisher’s exact test was used to explore qualitative variables as appropriate. The ROC curve was plotted using the R package “qROC.” [23] A heatmap was generated using the R package “gplots.” [24] If not specified above, *p* < 0.05 was considered statistically significant.

### Ethics statement

The TCGA is freely available to all investigators. Thus, supplementary approval by an ethics committee was not necessary in this study.

## Results

### Building and validation of survival-related gene set

**Fig. 1:**
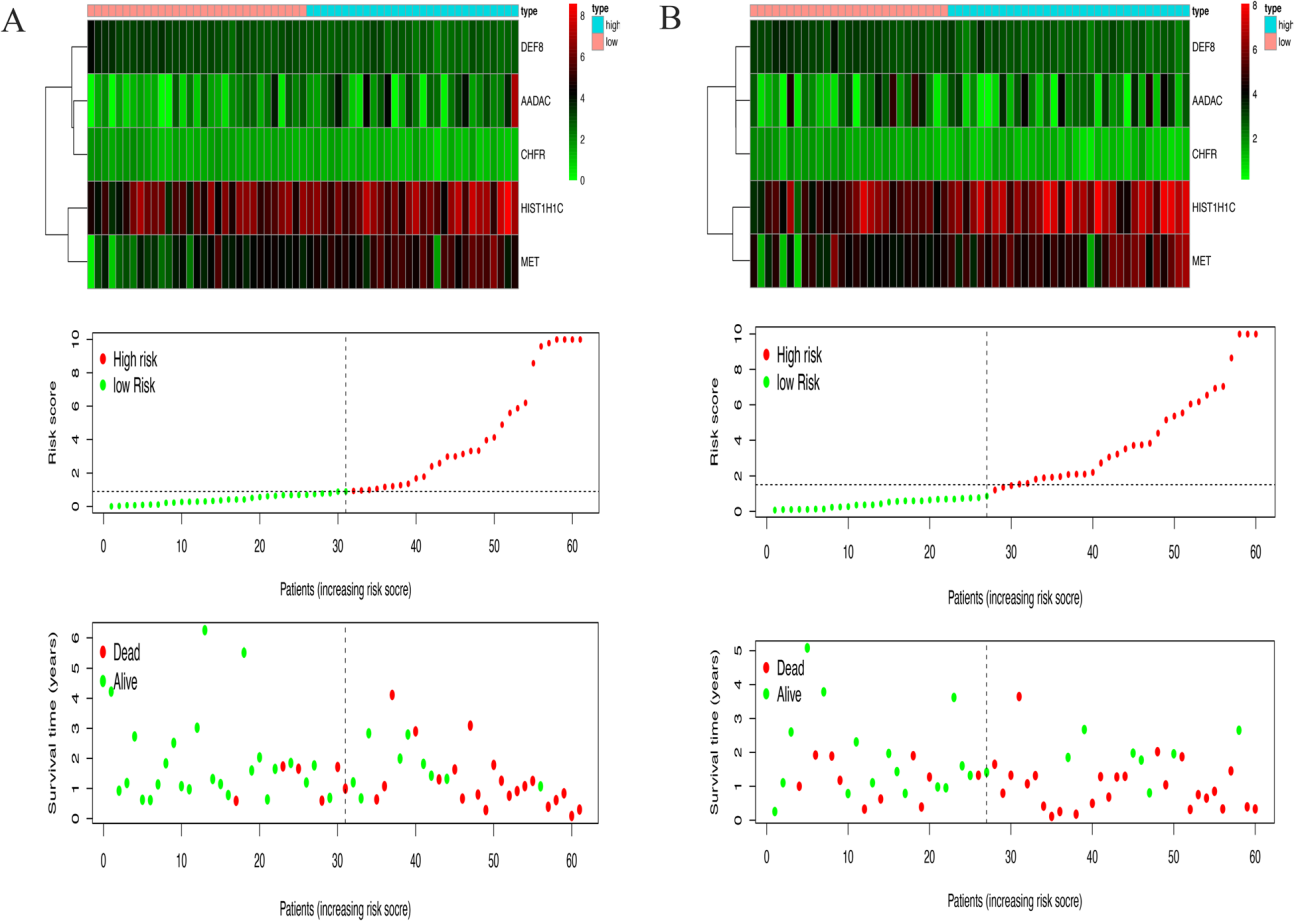
The heatmap of mRNA expression and a risk score analysis in the resectable pancreatic cancer respectively in primary set (**a**) and validation set (**b**)

One hundred twenty-one patients and 1055 genes were involved in the primary set to build the prognostic model. Univariable Cox regression, Lasso-penalized Cox analysis, and multivariable Cox analysis distinguished five genes for building a prognostic model. The five genes associated with the model were AADAC, DEF8, HIST1H1C, MET, and CHFR. The risk score was calculated as 0.022 × Expression of AADAC-0.320 × Expression of DEF8+ 0.007 × Expression of HIST1H1C + 0.041 × Expression of MET-0.989 × Expression of CHFR. The heatmap of mRNA expression and the risk score analysis for resectable pancreatic cancer were plotted for the primary set (Fig. 1a) and the validation set (Fig. 1b).

### Performance evaluation of risk score in the primary and validation set

**Fig. 2:**
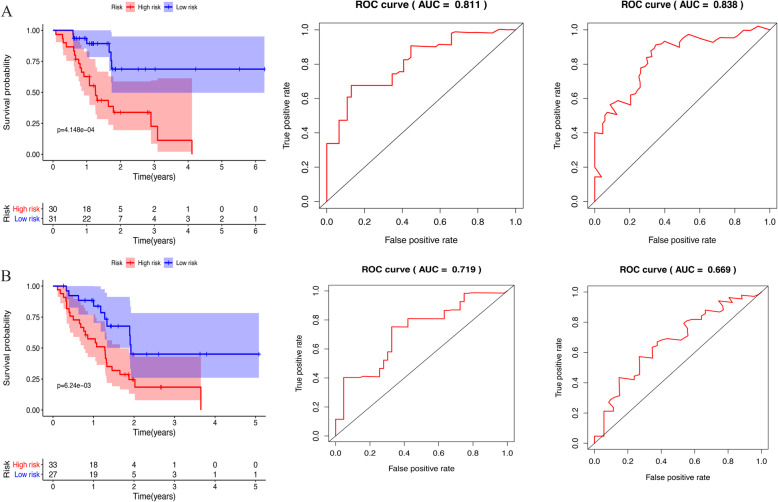
The Kaplan–Meier analysis and performance of risk score in predicting 1-and 3-year prognosis respectively in primary set (**a**) and validation set (**b**)

An optimal cutoff of 1.817 for the risk score was utilized to divide patients into a high-risk and a low-risk group in the primary set and the validation set. The overall survival of resectable pancreatic cancer based on risk score of the high-risk group was significantly more unfavorable than that of the low-risk group (*p* < 0.001; Fig. 2a). Additionally, the prognostic model was validated in the validation set. The overall survival of the resectable pancreatic cancer based on risk score of the high-risk group was significantly poorer than that of the low-risk group (p < 0.001; Fig. 2b). The AUC value calculated to evaluate the performance of the risk score for 1-and 3-year OS were 0.811 and 0.838 in the primary set (Fig. 2a) and 0.719 and 0.699 in the validation set (Fig. 2b).

### Evaluation of prognostic factors for resectable pancreatic cancer patients

One hundred and seventy-one patients were divided into a primary set and validation set with whole clinicopathological characteristics, including age, surgery method, radiation, gender, grade stage, and TNM stage, to build a second prognostic model (Table 1).

**Table 1:** Multivariate analysis of overall survival in TCGA

Variables		Primary set	Validation set			
		Multivariate analysis			Multivariate analysis	
	HR	95%CI	*P*-value	HR	95%CI	*P*-value
Age	1.051	1.006–1.099	0.024	1.003	0.965–1.041	0.871
Surgery method						
Other Method						
Whipple	2.208	1.414–3.499	0.004	1.182	0.739–1.903	0.491
Radiation						
Yes						
No	0.329	0.103–1.052	0.043	0.221	0.082–0.604	0.003
Gender						
MALE						
FEMALE	2.678	0.994–7.211	0.501	0.561	0.267–1.180	0.127
AJCC Stage						
I+ IIA						
IIB+ III+ IV	1.257	0.397–3.975	0.696	1.756	0.613–5.031	0.295
T Stage						
T1 + T2						
T3 + T4	1.149	1.042–2.302	0.436	3.348	1.148–9.759	0.026
N Stage						
N0						
N1	1.469	1.285–4.7444	0.499	1.573	0.076–4.322	0.998
Grade Stage						
G1						
G2	1.527	0.085–2.824	0.177	1.312	0.682–2.527	0.416
G3 + G4	2.297	1.217–4.355	0.610	2.510	1.279–4.928	0.707
Risk score	1.100	1.062–1.139	< 0.001	1.068	1.045–1.129	< 0.001

### Development and validation of the predictive nomogram

**Fig. 3:**
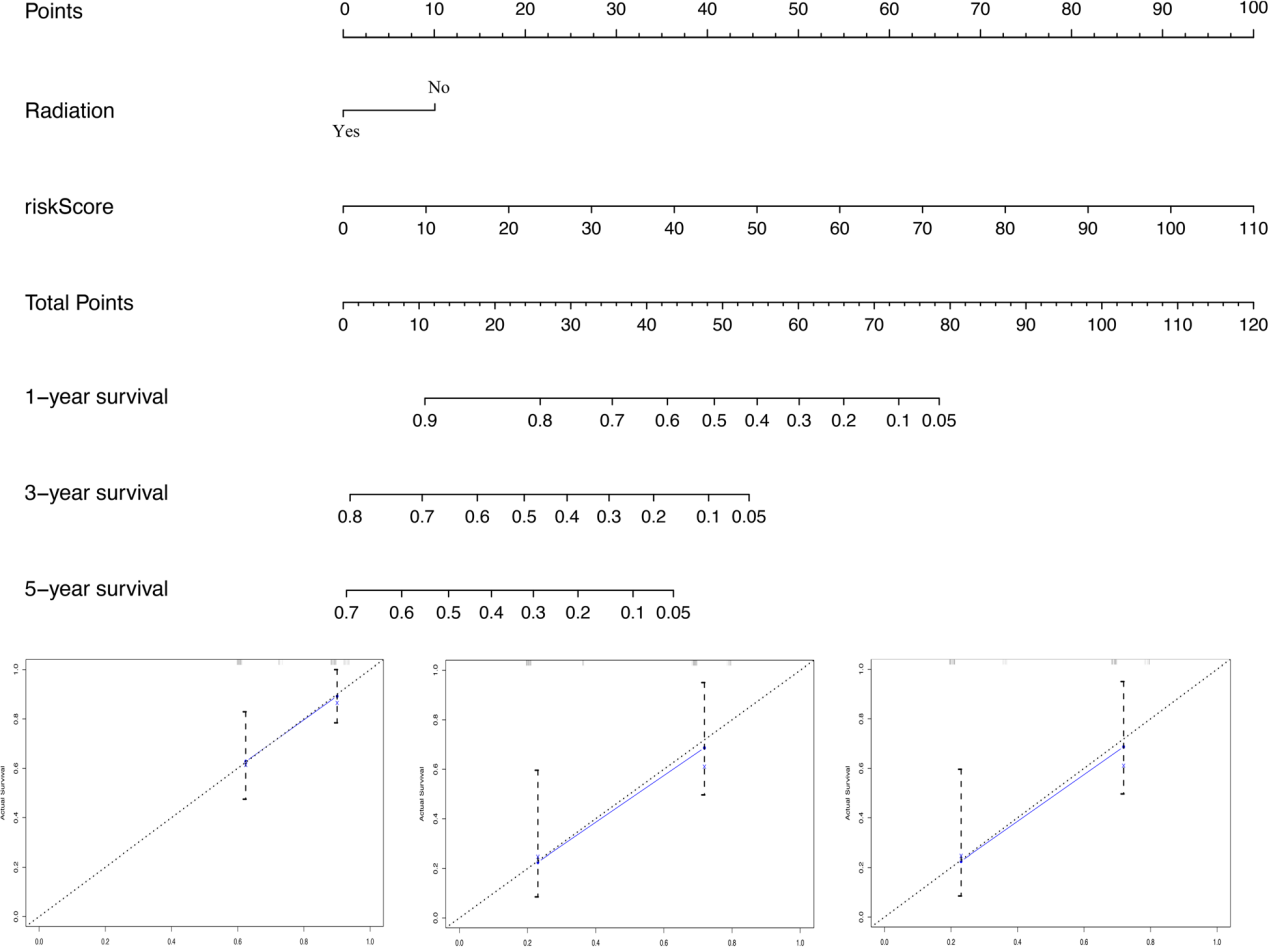
Nomogram for predicting 1-, 3-, and 5-year OS and Calibration curves for the nomogram in primary set

**Fig. 4:**
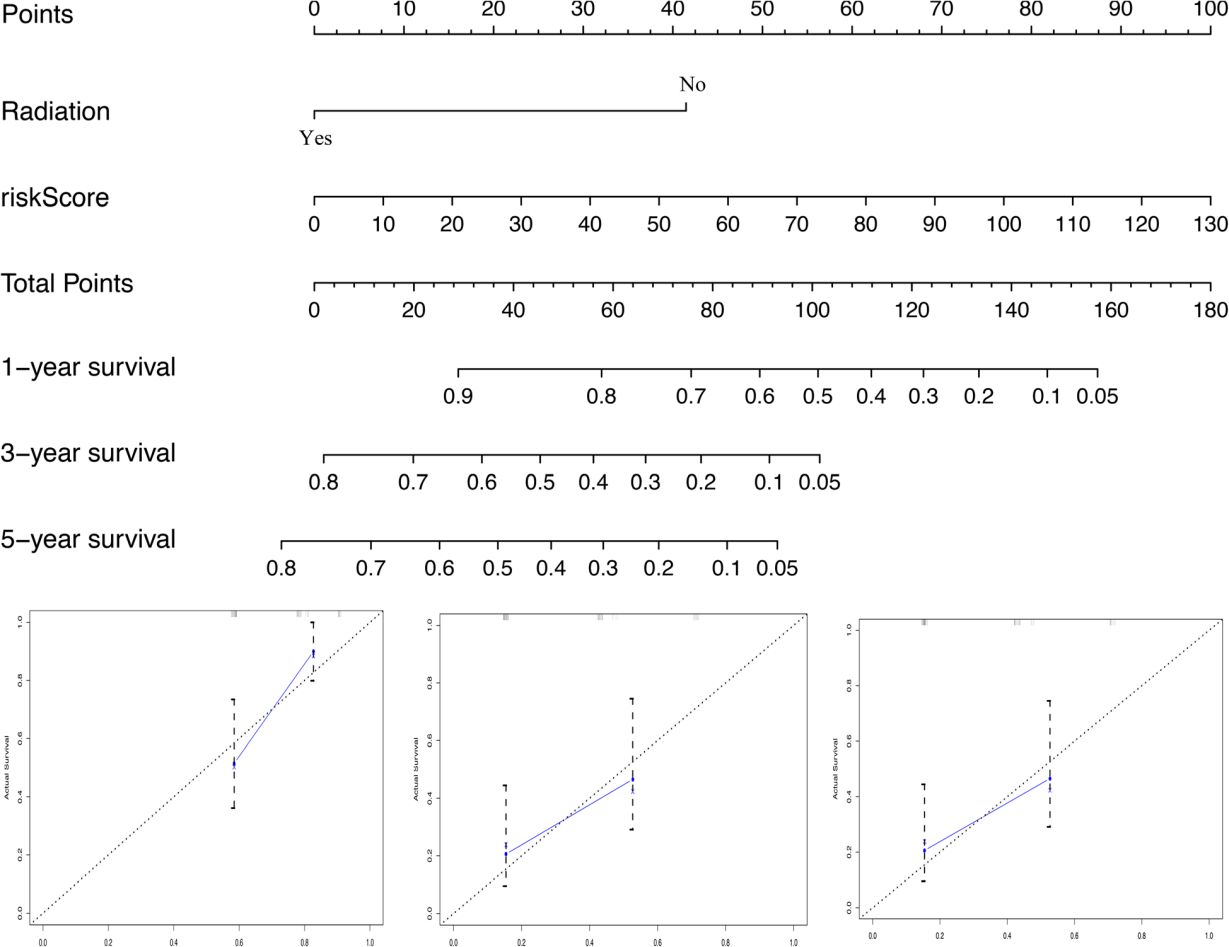
Nomogram for predicting 1-, 3-, and 5-year OS and Calibration curves for the nomogram in validation set

**Fig. 5:**
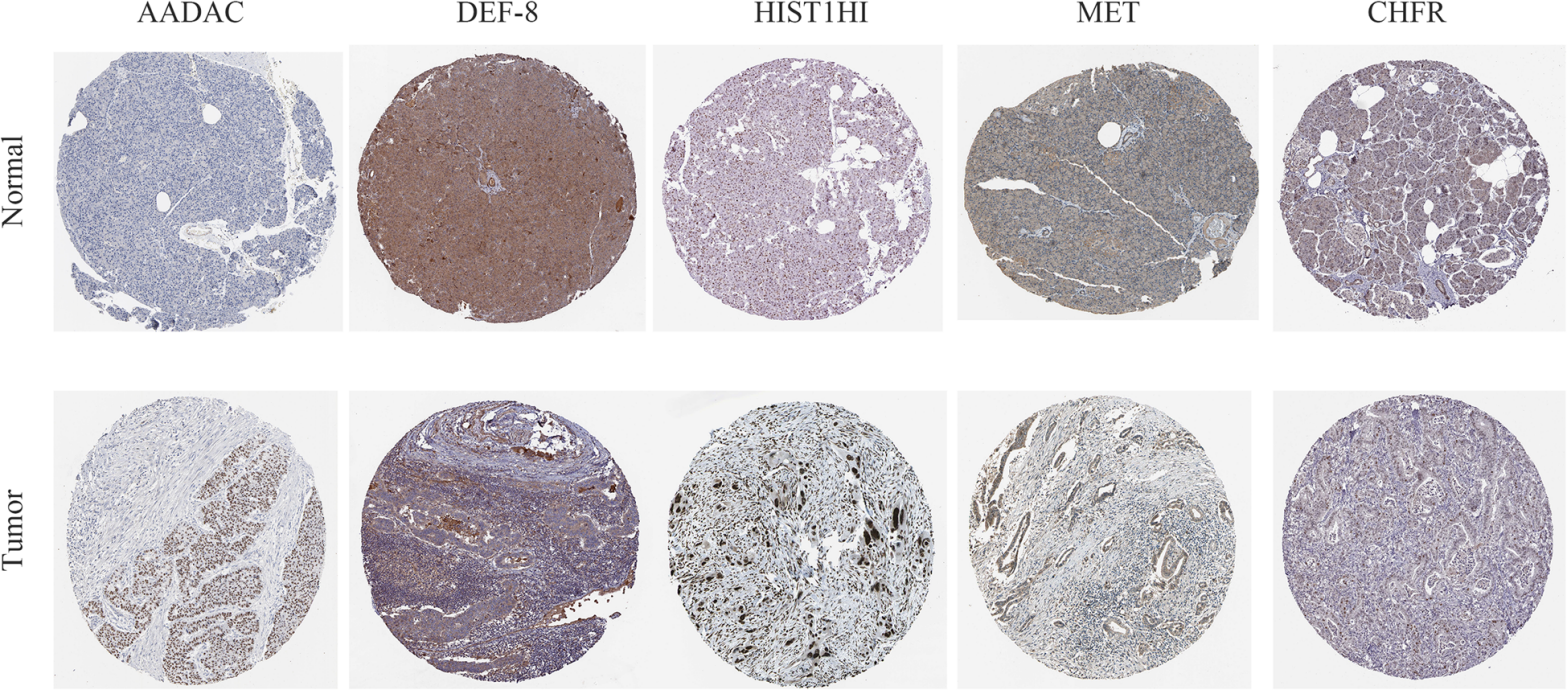
Validation of the protein expression of the five genes based The Human Protein Altas database (immunohistochemistry). The protein expression level of AADAC, DEF8, HIST1H1C, MET, and CHFR

The nomogram was built from radiotherapy-based characteristics and risk score for the primary set (Fig. 3a). The C-index [0.696 (0.608–0.784)], utilized to evaluate the predictive performance of the nomogram, indicated good performance for the primary set. The calibration curves for the nomogram showed that the nomogram model may be an ideal prediction model for the primary set (Fig. 3b). Meanwhile, the nomogram was validated for the validation set, and the corresponding calibration curves were generated (Fig. 4). The C-index [0.682 (0.606–0.758)] exhibited a greater predictive performance than the nomogram for the validation set. External validation using an online database showed that the proteins encoded by the DEF8 and CHFR genes were found to be significantly underexpressed, and those of the AADAC, HIST1H1C, and MET genes were found to be significantly overexpressed (Fig. 5). The patient immunochemistry data are listed in Supplementary Table S1.

### Gene set enrichment analyses

**Fig. 6:**
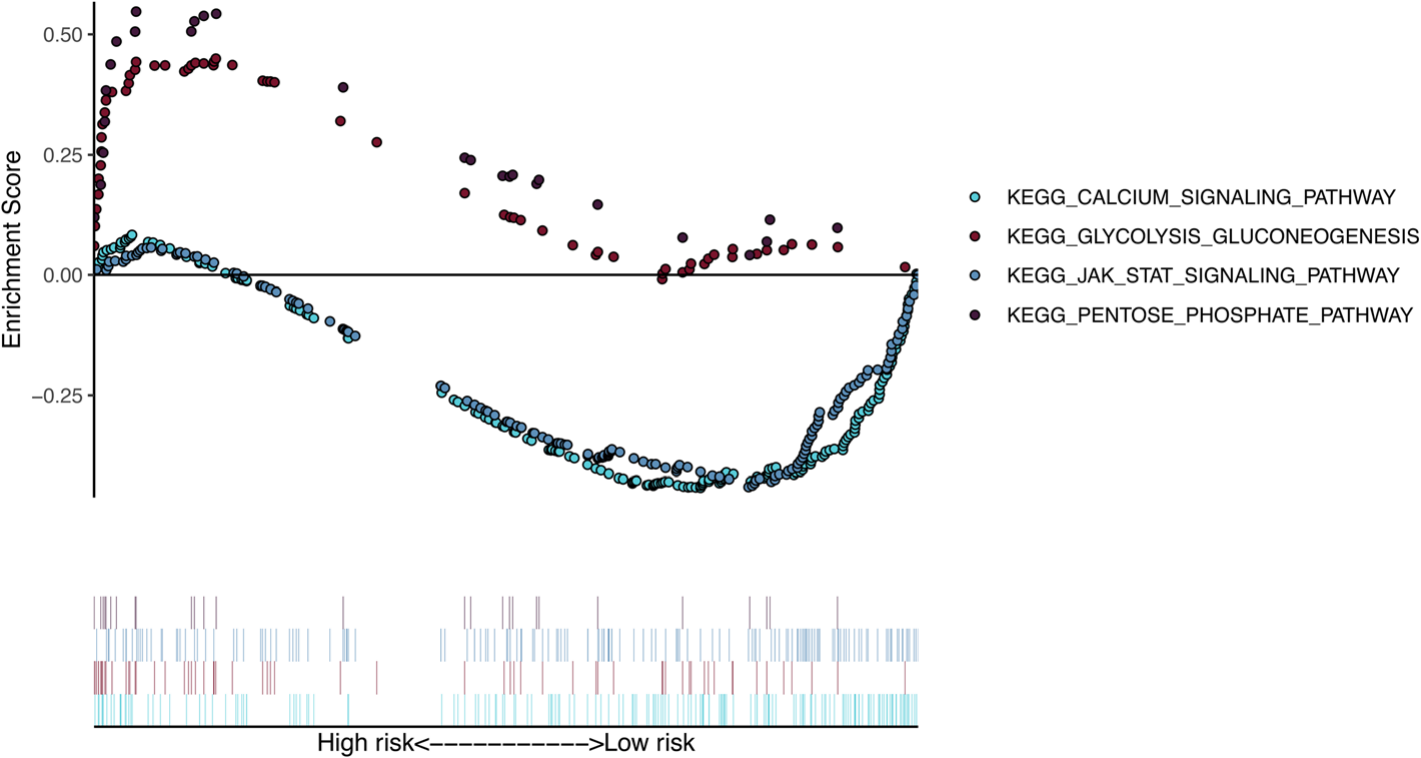
The significantly enriched KEGG pathways in by primary set or validation set by GSEA. Two representative KEGG pathways in high-risk patients and in low-risk patients. GSEA, Gene Set Enrichment Analyses

GSEA was implemented and identified 87 significantly enriched KEGG pathways. Among the enriched pathways involved were the calcium signaling pathway, JAK_STAT (The Janus kinase/signal transducer and activator of tranions) signaling pathway, and pentose phosphate signaling pathway. The others involved glycolysis gluconeogenesis-related physiological activity pathways commonly dysregulated in diseases (Fig. 6).

## Discussion

Pancreatic cancer has a dismal prognosis and is the seventh leading cause of cancer-related deaths worldwide [25, 26]. The diagnostic experiments available at present are nonspecific in the early stage, and most patients do not exhibit obvious symptoms in the advanced stage, leading to high mortality rates for the disease [27]. Thus, an analysis of aberrant genes as gene signatures in pancreatic cancer to reveal tumorigenesis and prognosis is meaningful. In particular, understanding aberrant genes that regulate molecular mechanisms in resectable PC may provide clinicians with new methods that can be utilized for the diagnosis and therapy of this disease. Recently, the dysfunction of mRNA was shown to occur in diverse cancers and is significantly involved in their prognoses [28–31]. For instance, some studies have revealed that mRNA gene signatures associated with certain characteristics such as the cell cycle or immune signature can be used in assessing the prediction of mortality risk in cancer [32, 33]. Unsurprisingly, many potential and worthy mRNAs would be indispensable to be identified to assess the prediction of mortality risk for resectable PC patients. Nevertheless, no explicit studies have discovered the aberrant gene expressions associated with prognosis in resectable PC patients. Thus, screening molecular biomarkers for resectable PC patients is urgently needed. In this study, we identified novel and useful SRGs that served as molecular biomarkers for resectable PC patients based on TCGA data.

Univariable Cox regression analysis, lasso-penalized Cox regression analysis, and multivariable Cox analysis suggested that five SRGs [AADAC (*P* = 0.008), DEF8 (*P* = 0.026), HIST1H1C(*P* = 0.031, MET(*P* = 0.001), and CHFR(*P* = 0.041)] were significantly associated with the prognosis of resectable PC,. The arylacetamide deacetylase gene (AADAC) lies on chromosome 3q25.1, and its expressed protein consists of 399 amino acids [34, 35]. The protein coded by AADAC protein is extensively implicated in the hydrolysis of various drugs [36], whose function may be related to chemotherapy resistance in pancreatic cancer. Previous experiments unveiled that the AADAC protein is expressed in the pancreas, adrenal glands, small intestine, and liver [37]. Differentially expressed in FDCP 8 homolog (DEF8) is a molecular component that modulates lysosome positioning and secretion [38]. Histone cluster 1 H1 family member c (HIST1H1C) is associated with modulating superior order chromatin structures and may be implemented to preserve DNA methylation patterns [39]. The overexpression of HIST1H1C is associated with unfavorable prognosis in adrenocortical carcinoma [40] and nonfunctional pituitary adenomas [41]. Methyltransferase (MET) is a proto-oncogene encoding the receptor tyrosine kinase c-MET for hepatocyte growth factor (HGF) [42], which triggers cell migration, proliferation, and angiogenesis. Aberrant MET expression is commonly expressed in various malignancies [43–47]. The protein coded by CHFR (Checkpoint with fork head and ring finger domains) serves as a checkpoint that might play diverse roles at different phases of the cell cycle [48, 49]. Specifically, there is evidence that downregulation of CHFR performs this checkpoint function in pancreatic cell lines [50]. The downregulation of CHFR is also correlated with poor prognosis in lung cancer [51], colon cancer [52], and gastric cancers [53]. Some previous experiments showed that targeting CHFR in cancer therapy is effective. For instance, the downregulation of CHFR in Oral squamous cell carcinoma cells was found to be effective in increasing the response to docetaxel [54]. Similarly, the downregulation of CHFR in gastric cancer patients makes them more sensitive to docetaxel exposure [55]. Consequently, CHFR might be a molecular target of therapy in the future. Noteworthy, Henriksen and colleagues disclosed that CHFR gene methylation was involved in lymph node metastasis in patients with PC [56].

We revealed many significantly enriched pathways for the five gene signatures in GSEA, one of which is the JAK-STAT signaling pathway. The JAK-STAT signaling pathway is principally involved in cytokines and growth factors. JAK initiation irritates cell proliferation, differentiation, migration and apoptosis in mammals [57], and previous experiments suggest that this pathway is correlated with pancreatic cancer [58]. As described above, the five gene signatures are associated with dysregulated signaling pathways that may serve as potential molecular targets for therapy for resectable PC.

This study found that the risk score based on prognosis-related genes had a relatively excellent and consistent performance in predicting OS in resectable PC. The AUC based on the risk score for 1-year survival was more than 0.70, indicating a relatively high diagnostic performance in the primary dataset and validation set. For 3-year survival, the AUC based on the risk score was 0.699, indicating a relatively low diagnostic performance in the validation set. However, when the gene signatures alone were used to build a predictive model, it was unable to adequately predict the prognosis of resectable pancreatic cancer. For this reason, we integrated clinicopathological data and gene information to build a predictive nomogram. The C-index [0.696 (0.608–0.784)], utilized to evaluate the predictive performance of the nomogram, indicated good performance for the primary set. Additionally, the C-index [0.682 (0.606–0.758)] exhibited a relatively greater predictive performance by the nomogram for the validation set. Our predictive model suggests that resectable pancreatic cancer patients may benefit from postoperative radiotherapy. External validation using an online database showed that the proteins coded by DEF8 and CHFR were found to be significantly underexpressed and those coded by AADAC, HIST1H1C, and MET were found to be significantly overexpressed (Fig. 5). Thus, our predictive nomogram may contribute to the assessment and development of therapeutic decisions for resectable PC patients.

Few limitations of our studies are as follows: First, we used a single set to build a predictive nomogram. Therefore, a more independent and external set is necessary to verify the predictive ability of the nomogram. Second, functional experiments are necessary to determine the intangible mechanisms of the predictive genes.

## Conclusions

Our study identified five prognostic gene biomarkers for OS prediction and might facilitate postoperative molecular targeting therapy for resectable PC. In particular, the genomic-clinical nomogram may be an effective model for evaluating postoperative OS and developing an optimal therapeutic schedule for patients with resectable PC.

## Supplementary information

**Additional file 1.** Supplementary Table S1. Clinical Traits of Immunohistochemistry in the Human Protein Atlas Database.

**Additional file 2.**

**Additional file 3.**

## Data Availability

The primary set data in Supplementary S2 and the validation set data are listed in Supplementary S3.

## Abbreviations

PC: Pancreatic cancer
TCGA: The cancer genome atlas
SRGs: Survival-related genes
OS: Overall survival
KEGG: Kyoto encyclopedia of genes and genomes
C-index: Concordance index
AUC: Area under the curve
MET: Methyltransferase
CHFR: Checkpoint with fork head and ring finger domains
AADAC: Arylacetamide deacetylase gene
DEF8: Differentially expressed in FDCP 8 homolog\HIST1H1C Histone cluster 1 H1 family member c
GSEA: Gene set enrichment analyses
JAK_STAT: The janus kinase/signal transducer and activator of tranions
