# Supplementary material for: Five gene signatures were identified in the prediction of overall survival in resectable pancreatic cancer
Source: BMC Surg. 2020 Sep 17;20:207. doi: 10.1186/s12893-020-00856-y (PMC7499920; doi:10.1186/s12893-020-00856-y)
Supplement: Supplementary file 1 — Additional file 1. Supplementary Table S1. Clinical Traits of Immunohistochemistry in the Human Protein Atlas Database. [file 12893_2020_856_MOESM1_ESM.pdf]

Supplementary Table S1. Clinical Traits of Immunohistochemistry in the Human Protein Atlas Database

Gene symbol	Tissue	ID	Age	Gender	Staining	Intensity	Quantity
AADAC	Normal	2032	35	Female	Low	weak	75%–25%
	Tumor	1904	50	Male	Medium	Moderate	>75%
DEF8	Normal	2940	36	Male	Medium	Medium	>75%
	Tumor	3614	61	Female	Low	weak	>75%
HIST1H1C	Normal	3219	35	Male	High	Strong	>75%
	Tumor	64	73	Male	High	Strong	>75%
MET	Normal	2220	43	Female	Low	weak	>75%
	Tumor	977	57	Female	Medium	Moderate	>75%
CHFR	Normal	5075	58	Female	Medium	Moderate	75%–25%
	Tumor	4400	71	Male	Low	Moderate	<25%
